# Ecofriendly magnesium oxide nanoparticles: anticancer, antimicrobial, and antidiabetic potentials in vitro

**DOI:** 10.1186/s13568-025-01950-1

**Published:** 2025-10-03

**Authors:** Mohamed K. Y. Soliman, Adel Hussain Talib, R. Mahmoud, Zainab Anwar Ali, Halah H. Al-Haideri, Adil Abalkhail, Abdulkarim S. Binshaya, Mai Hamed Salem, Fatimah O. Al-Otibi, Mohamed Taha Yassin

**Affiliations:** 1 Botany and Microbiology Department, Faculty of Science, Al-Azhar University, Nasr City, 11884 Cairo Egypt; 2 Department of Biology, College of Science for Women, University of Baghdad, Baghdad, 10072 Iraq; 3 Department of Pharmacology, Toxicology and Supporting Science, College of Pharmacy, Al-Farahidi University Baghdad, Baghdad, Iraq; 4 Department of Public Health, College of Applied Medical Sciences, Qassim University, P.O. Box 6666, 51452 Buraydah, Saudi Arabia; 5 Department of Medical Laboratory, College of Applied Medical Sciences, Prince Sattam Bin Abdulaziz University, 11942 Alkharj, Saudi Arabia; 6 Department of Microbiology and Immunology, College of Pharmaceutical Sciences and Drug Manufacturing, Misr University for Sciences and Technology, P.O. Box 77, Giza, Egypt; 7 Department of Botany and Microbiology, College of Science, King Saud University, 11451 Riyadh, Saudi Arabia; 8 King Salman Center for Disability Research, 11614 Riyadh, Saudi Arabia

**Keywords:** MgO NPs, Anticancer, Antibacterial, Antioxidant, Antidiabetic, Disability, Public health

## Abstract

This research on bioinspired magnesium oxide nanoparticles (MgO NPs) synthesized using orange peel extract (OPE) holds significant promise for improving the health and quality of life of individuals with disabilities. Phytochemical analysis for OPE was performed using recommended standard procedures, which confirmed that OPE served as a rich source of various essential phytochemicals. Response surface methodology was employed to optimize MgO synthesis and maximize nanoparticle yield. The results obtained from characterization (Uv, XRD, TEM, SEM, Zeta potential, EDX, DLS and FTIR) indicated the semispherical or quasi-spherical shape of MgO NPs with 53.3 nm in the mean size. The MgO NPs displayed antioxidant features, with the value of IC_50_ 180.2 μg/mL. Additionally, MgO NPs had cancer therapy against the HeLa cervical cancerous cell line and (PC3) prostate cancerous cell line, where the IC_50_ was 179.4 µg/mL and 190.67 µg/mL correspondingly, while showing less toxicity toward the normal Vero cell line. The biosynthesized MgO NPs displayed significant antibacterial activity. The largest zone of inhibition was against *Bacillus subtilis *(27.57 mm, MIC 62.5 µg/mL), followed by *Staphylococcus aureus* (18.4 mm, MIC 62.5 µg/mL), *Salmonella typhi* (18.0 mm, MIC 125 µg/mL), and *Klebsiella pneumoniae* (16.8 mm, MIC 250 µg/mL). They also showed anti-biofilm activity against *S. aureus* ATCC 35556 and *E. coli* ATCC 25922 with inhibition of 69.6 and 55.5% respectively at 200 μg/mL. In addition, Biosynthesized MgO NPs demonstrated significant inhibitory activity against α-amylase as well as α-glucosidase enzymes, with IC_50_ 76.71 μg/mL and 38.8 μg/mL, respectively.

**Graphical abstract:**

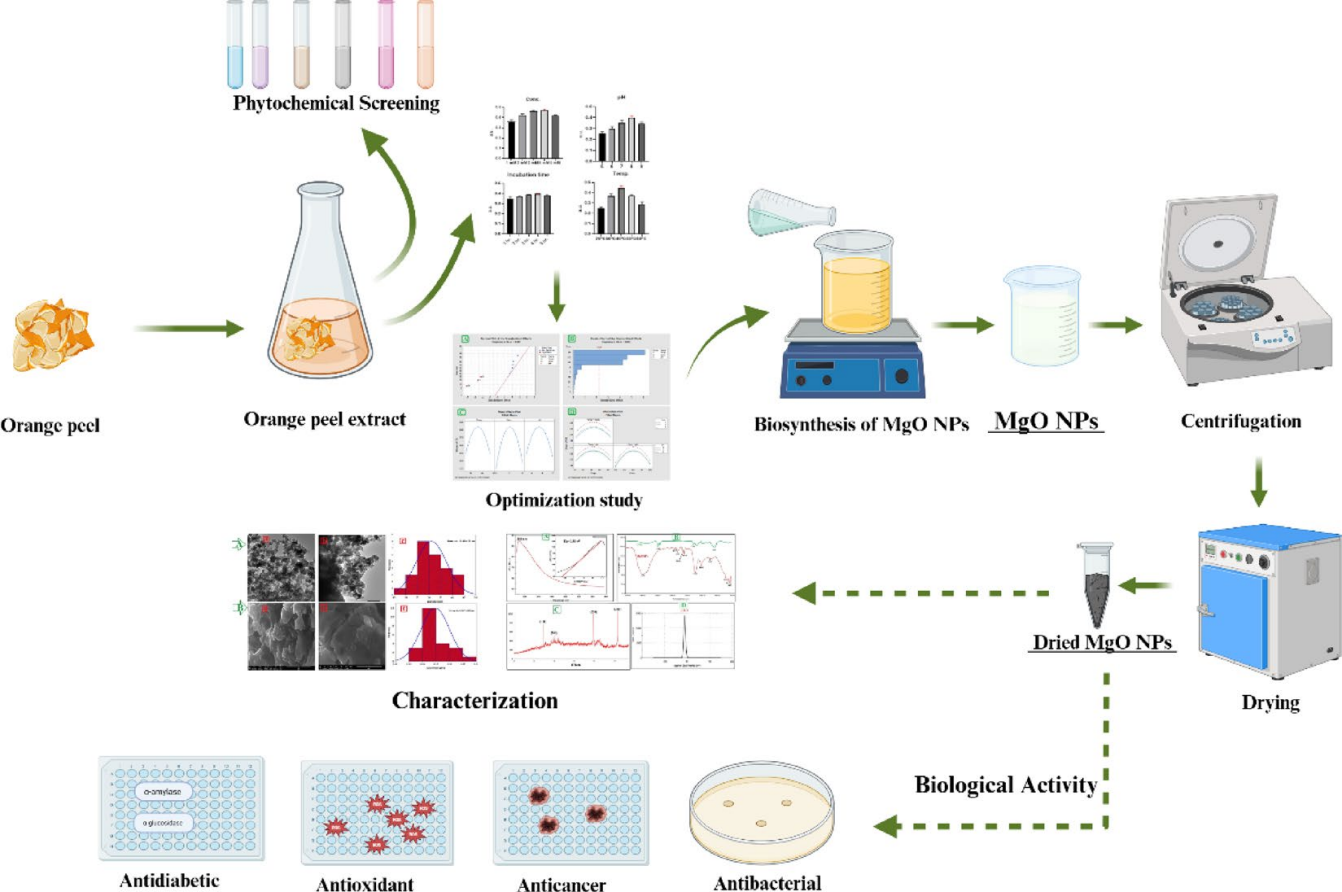

**Supplementary Information:**

The online version contains supplementary material available at 10.1186/s13568-025-01950-1.

## Introduction

Nanomaterials are distinguished by their nanosized dimensions, which impart better mechanical and electrical characteristics than macroscopic bulk particles (Abdal Dayem et al. 2017; Chan et al. 2025; Cheah et al. 2025; Soliman and Salem 2025; Kumar Chandraker et al. 2022b). In recent years, this field has gained significant interest due to its broad applicability across multiple domains, including catalysis, gas sensing, energy storage, and energy conversion technologies (Sasidharan et al. 2024; Liang et al. 2025). Two main strategies, known as top-down and bottom-up approaches, are commonly employed for the fabrication of nanomaterials (Kaur et al. 2024; Kumar Chandraker et al. 2022a; Soliman et al. 2024a, 2023). Bio-inspired synthesis has recently emerged as a sustainable pathway for producing nanomaterials (Chandraker et al. 2019b; Wong et al. 2024). Bio-extracts, which are rich in biomolecules such as alkaloids, terpenoids, and polyphenols, possess functional groups that efficiently bind to metal ions. Moreover, they contribute to the reduction of particle size by modulating the nucleation and growth phases during nanoparticle formation (Chandraker et al. 2022c; Baqer et al. 2025; Selim et al. 2025; Shaikh et al. 2021). Multiple reports state that a large number of recent publications concentrate on creating nanoparticles using reducing agents derived from plant extracts (Chandraker et al. 2019a, 2021, 2020; Mehra et al. 2025; Almuhayawi et al. 2024; Soliman et al. 2024c). The potent broad-spectrum antibacterial and antibiofilm activity of nanoparticles offers a novel approach to combatting infections (Chandraker et al. 2022b) and several pathogenic microbes prevalent in populations with mobility impairments or compromised immunity (Subramaniam et al. 2024; Chan et al. 2024; Chandraker et al. 2022a). Furthermore, their demonstrated anticancer activity against specific cell lines, robust antioxidant effects to mitigate oxidative stress, and strong antidiabetic potential via enzyme inhibition address critical comorbidities and secondary conditions disproportionately affecting people with disabilities (Vamsi Krishna et al. 2024; Al-Harbi et al. 2024).

Magnesium oxide (MgO) has received widespread attention for its multifaceted role in biomedical research. Commonly found as a white solid mineral, this inorganic compound possesses distinct properties that make it highly relevant to a broad range of scientific disciplines, including medicine, biochemistry, physiology, and nutrition (Chinthala et al. 2021).The significance of MgO primarily stems from magnesium itself, a vital mineral that is necessary for many physiological functions in living systems. Magnesium functions as a vital cofactor in a wide array of enzymatic reactions, contributes to the regulation of ion channels, and is integral to the structural stabilization of nucleic acids as DNA and RNA. The formation of MgO through the reaction of magnesium with oxygen produces a compound with notable potential in supporting cellular functions, maintaining tissue integrity, and promoting overall organismal health (Seitz et al. 2014). The distinctive microstructural features of MgO, such as its large surface area, high pore volume, and the presence of both acidic and basic active sites, enhance its surface reactivity across diverse applications (Yi et al. 2014). Additionally, the varied morphologies of MgO including rod-shaped, spherical, platelet-like, floral, star-shaped, cubic, and needle-like structures make it a promising candidate for the emergence of nanomaterials with innovative and exceptional physicochemical properties (Abdallah et al. 2019). There has been a marked increase in scholarly publications focusing on the advancements and potential applications of MgO NPs. Extensive research has demonstrated their effectiveness in areas such as photocatalysis, antimicrobial activity, and integration into energy storage systems and sensing technologies. Recently, there has been growing interest in investigating MgO NPs within the field of biomedical engineering. Their versatility has enabled their application in various biomedical contexts, including tissue repair, implant surface coatings, diagnostic imaging, wound management, and the development of anticancer therapies (Wahid et al. 2020). Combined with their relatively low toxicity to normal cells and sustainable synthesis, these multi-functional MgO NPs represent a valuable platform for developing targeted therapeutic strategies (Gatou et al. 2024; Hetta et al. 2023).

Cervical and prostate cancers pose significant public health concerns on a global scale, contributing markedly to cancer-related deaths. Cervical cancer is recognized as the fourth most commonly diagnosed cancer among females worldwide. According to the World Health Organization (WHO), an estimated 604,000 new cases of cervical cancer were reported in 2020, with approximately 342,000 associated fatalities (Singh et al. 2022). Contrarily, prostate cancer is the second most common disease diagnosed globally (Bhattacharyya et al. 2004). According to the American Cancer Society, roughly 34,130 people died from prostate cancer in 2021, and there were about 248,530 new cases diagnosed in the USA alone (Al-Fayez and El-Metwally 2023). Prostate and cervical malignancies provide substantial challenges for a number of reasons. The high death rates linked to these malignancies are caused by a combination of factors, including delayed diagnosis, limited availability of screening and early detection tools, socioeconomic disparities, and insufficient public awareness (Hamdi et al. 2021; Wang et al. 2010). The available treatments for these cancers frequently have drawbacks, such as adverse reactions and the emergence of drug resistance (Majumder et al. 2019). Considering the elevated prevalence of prostate cancer in men and cervical cancer in women, there is a pressing need to explore novel therapeutic approaches that can effectively target and manage these malignancies. Beyond the demonstrated biomedical potential, the eco-friendly synthesis and multifunctional properties of these orange peel-derived MgO NPs suggest promising environmental applications. The green synthesis approach itself represents a significant environmental advantage by utilizing agri-waste (orange peel), reducing reliance on hazardous chemicals, and minimizing energy consumption and waste generation compared to conventional physico-chemical methods (Alsaiari et al. 2023).

This research on eco-friendly MgO NPs holds profound significance for the health and well-being of individuals with disabilities. This population experiences well-documented health disparities, including a higher susceptibility to antimicrobial-resistant infections due to frequent medical interventions and compromised immunity (Hetta et al. 2023), a greater risk of pressure injuries and chronic wounds from immobility (Mirhaj et al. 2022), and an increased prevalence of comorbid conditions like type 2 diabetes (Robertson et al. 2015). The multifunctional therapeutic potential of these orange peel-derived MgO NPs directly addresses these complex needs. Their potent broad-spectrum antibacterial and antibiofilm activity offers a novel, nano-based strategy to combat infections and promote wound healing, crucial for populations with mobility impairments. Furthermore, the demonstrated anticancer activity against cervical and prostate cell lines, robust antioxidant effects, and strong antidiabetic potential via enzyme inhibition collectively target critical comorbidities that exacerbate disability. By providing a single, green-synthesized agent with this multi-target efficacy, the study aligns with the urgent need for innovative therapeutic strategies that can reduce polypharmacy and improve the overall quality of life for disabled individuals (Vagena et al. 2024).

In our investigation, orange peel extract was utilized for the biological synthesis of MgO NPs. Response surface methodology (RSM) was employed to optimize three significant factors: pH, concentration, and temperature. Then characterize these NPs by Uv, Zeta potential, TEM, FTIR, EDX, XRD, SEM–EDX and DLS. The bio-derived MgO NPs were investigated for their anticancer efficacy, antibacterial and antibiofilm potential, antioxidant activity and as an antidiabetic agent.

## Materials and methods

### Orange peel extract (OPE) preparation

OPE was sourced from local market (BIM) in Cairo City, Egypt and transported to the laboratory for processing. OPE from fruits without any visible plant disease symptoms then washed via DH_2_O. All the peels were cut into tiny pieces not more 1 cm for each piece, and 200 g was mixed with 1 L of deionized H_2_O. The mixture was blended at 1000 rpm for about 3 min, filtered through filter paper, and the gathered extract was put in a clean bottle. The prepared OPE was stored at 4 °C until further use (Salem et al. 2022).

### Phytochemical screening of OPE extract

The active constituents in this study were identified using standard, widely accepted phytochemical screening methods. The presence of various phytochemicals was confirmed using established qualitative assays: Wagner’s reagent for alkaloids; Molisch’s test for carbohydrates; Keller-Killiani’s test for cardiac glycosides; alkaline reagent test for flavonoids; ferric chloride test for phenols; precipitation test for phlobatannins; ninhydrin test for proteins; foam test for saponins; Liebermann-Burchard test for steroids; Braymer’s test for tannins; Salkowski’s test for terpenoids; hydrochloric acid (HCl) test for quinones; and glacial acetic acid test for oxalates (Akinlabu et al. 2024; Owoeye et al. 2023).

### Optimization study

#### Optimization assessment of MgO NPs biosynthesis (general factors)

The characteristics related to the OPE and magnesium nitrate, its main nanoparticle component, were adjusted through optimization studies. The impact of different filtrate and precursor solution durations (1.0, 2.0, 3.0, 4.0, and 5.0 h), precursor concentrations (1.0, 2.0, 3.0, 4.0, and 5.0 mM), pH value (5, 6, 7, 8, and 9), and temperature (20, 30, 40, 50 and 60 °C) were subsequently evaluated using the one-variable-at-a-time (OVAT) approach (Khan et al. 2020).

#### Statistical optimization using central composite design (CCD)

Statistical optimization was conducted using RSM. Among the various RSM techniques, the CCD is one of the most widely used and effective approaches for optimization. CCD enables the evaluation of relationships between independent variables and the response in this case, precursor concentration, pH, and temperature. Within the experimental framework, cube points are utilized to model first-order linear effects and interactions, axial points to estimate second-order quadratic effects, and center points to evaluate the curvature of the response surface and determine pure experimental error. Output from the CCD, both numerical and graphical, facilitate the generation of optimization curves that guide the identification of optimal variable settings to enhance the synthesis of MgO NPs. In this study, a two-level, three-factor CCD model was employed. A full factorial CCD was implemented to optimize MgO NPs production. Each response variable obtained from the CCD experiments was modeled using a full quadratic (second-order polynomial) multiple regression approach, considering three continuous independent variables (precursor concentration, pH, and temperature) across various levels. The statistical significance was caaried out via ANOVA foe evaluation the model terms, including linear, quadratic (squared), and interaction effects among the variables (Salem et al. 2024).

#### Validation of the RSM model

The response optimizer in the Minitab® DoE package was employed to generate optimization curves and identify the optimal combination of interacting factors for maximizing MgO NP production. The optimization process was guided by individual desirability (d) and composite desirability (D) functions, which evaluate how effectively the predicted settings meet the desired response outcomes on a scale from 0 to 1. The validation was carried via the prediction model, additional confirmation study was conducted under the optimized conditions. All experimental design, data generation, and the Minitab® version 18 (2017) program was used for statistical analyses, supplemented with built-in statistical and graphical analysis tools.

#### Biosynthesis of MgO NPs at optimum condition

MgO NPs were synthesized by a green, eco-friendly route utilizing OPE. Specifically, OPE was combined with 5.1 mM magnesium nitrate (Magnesium nitrate hexahydrate (Mg(NO_3_)_2_·6H_2_O ≥ 99.0%, Sigma-Aldrich, St. Louis, MO, USA) solution (1:10), which served as the magnesium source. The mixture was prepared at a pH of 6.8 and maintained at a temperature of 32.5 °C. A control setup was prepared using 10 mL of distilled water instead of OPE under identical conditions. Both reaction mixtures were shaken in the dark on a rotary shaker for 3 h to ensure thorough mixing. The resulting MgO NPs were isolated and purified through repeated washing with DH_2_O followed by centrifugation. The resulting NPs were dried in a hot air oven at 80 °C for 12 h to obtain a dry powder and stored at ambient temperature for subsequent characterization and analysis (Jain et al. 2019; Salem et al. 2022). To ensure reproducibility, the synthesis was performed in triplicate (n = 3) under identical optimized conditions.

#### Characterization studies of bio generated MgO NPs

During the incubation process, the biofabrication of MgO NPs was preliminarily evidenced by an observable color change in the reaction mixture. Ultraviolet–visible (UV–Vis) spectrophotometry was employed to monitor the nanoparticle formation using a JENWAY-6305 spectrophotometer across a wavelength range of 200–800 nm. The functional groups on the surface of the synthesized MgO nanoparticles were characterized using FTIR spectroscopy (Perkin-Elmer Spectrum Two, USA). The spectrum range was started from 400 to 4000 cm^−1^ with a resolution of 4 cm^−1^, averaging 32 scans to enhance signal quality. XRD analysis (Diano diffractometer, Philips, CuKα radiation, λ = 0.15418 nm, 45 kV) was performed to verify the crystalline structure of the nanoparticles. The colloidal stability of the biosynthesized MgO NPs was assessed by measuring zeta potential using a Zetasizer Nano ZS analyzer (Malvern) (Kuruthukulangara and Asharani 2024). Morphological and size analysis was conducted using TEM (JEOL-2010, Japan), while surface characteristics and particle distribution were examined via SEM (ZEISS EVO-MA 10, Germany). Elemental composition and purity assessment was performed using energy-dispersive X-ray spectroscopy (EDX, BRUKER, Germany) coupled with SEM (Soliman et al. 2024b; Suliman et al. 2025). Additionally, DLS was employed to assess particle size distribution, with nanoparticles dispersed in Milli-Q water to minimize interference from scattering artifacts (Fouda et al. 2023).

#### Anticancer activity

The study evaluated two ATCC cancer cell lines HeLa (cervical carcinoma) and PC3 (prostate carcinoma) alongside normal Vero cells. Cells were seeded at 1 × 10^5^ cells/mL (100 µL/well) in 96-well plates and incubated for formation monolayer for about 24 h at approximately 37 °C. After removing the culture medium, the monolayer was rinsed twice with washing buffer to remove debris. Test samples, serially diluted in serum-supplemented RPMI (2% serum) to achieve a final concentration range of 2000 to 62.5 μg/mL, were added to the wells (0.1 mL/well), with three wells serving as untreated controls (maintenance medium only). Post-incubation, cytotoxicity was assessed microscopically for morphological changes such as cell rounding, detachment, granulation, or monolayer disintegration. For the MTT (≥ 97.5% purity Sigma-Aldrich St. Louis, MO, USA) assay, viable cells metabolized MTT (5 mg/mL in PBS) into formazan, which was dissolved in 200 µL DMSO with 5 min of shaking (150 rpm). Absorbance at 560 nm, measured via microplate reader, correlated directly with cell viability, enabling quantitative cytotoxicity analysis (Soliman et al. 2024c; Tolosa et al. 2014).

#### Antibacterial and antibiofilm efficacy

Agar-based well diffusion technique was used to assess how successfully green generated MgO NPs inhibited the development of harmful bacteria (Saied et al. 2025; Balouiri et al. 2016). The potential of synthesized MgO NPs as an antibacterial agent was assessed towards selected pathogenic strains, comprising *Klebsiella pneumonia* (ATCC 13883), *Bacillus subtilis* (ATCC 6633) and *Staphylococcus aureus* (ATCC 6538) and *Salmonella typhi* (ATCC 6539). Each strain was sub-cultured on nutrient agar (Oxoid Ltd., Basingstoke, Hampshire, UK) and incubated at (37 °C/24 h). A single colony from each culture was aseptically collected using a sterile swab and uniformly spread onto Muller–Hinton agar plates. Three wells (6 mm) were then carefully punched into each inoculated plate. Each well was loaded with 100 μL of MgO nanoparticle suspension at a concentration of 1000 μg/mL. Following incubation at optimum condition (37 °C/24 h), the evaluation of antibacterial efficacy via measuring the zone the inhibition formed around each well (Perez 1990). Antibacterial activity was assessed using triplicate independent experiments for each strain.

MIC of MgO NPs towards the microbial strains was assessed using the broth microdilution technique, following the CLSI guidelines. Two-fold serial dilutions of MgO NPs were prepared to obtain seven concentrations levels (1000 to 15.75 μg/mL). Sterile 96-well microtiter plates were filled with 100 μL of Mueller–Hinton broth (Oxoid) per well. An additional about 100 μL of level dose of MgO NPs was introduced to the wells. Subsequently, 20 μL of a bacterial suspension calibrated to the 0.5 McFarland standard was introduced into each well, except for the negative control, which contained only broth and nanoparticles. Positive control wells were supplemented with bacterial suspension in broth without nanoparticles to confirm microbial viability and medium suitability. The plates were incubated at (37 °C/ 24 h.). After 24 h, the microbial growth was assessed using a microplate reader (STATFAX, USA) (Abbey and Deak 2019).

The MgO NPs antibiofilm efficacy toward *S. aureus* ATCC 35556 and *E. coli* ATCC 25922 a biofilm producer strains were examined using an adapted microtiter plate assay. Various doses of MgO NPs (3.12–200 µg/mL) were added to broth bacterial strains standardized to 0.5 McFarland and diluted 1:100 in glucose-supplemented tryptic soy broth (Oxoid) within 96-well plates. After 48 h at 37°C of incubation, all of the planktonic cells were eliminated, and the wells were gently washed with PBS (pH 7.4). The established biofilms were chemically fixed using methanol (95% v/v) and subsequently stained with crystal violet solution (0.3% w/v) for 15–20 min under ambient conditions. After that washing with deionized water to remove unbound stain, the adherent biofilm matrix was destained with acetic acid solution (30% v/v). Quantitative analysis of biofilm formation was performed by measuring optical density at 540 nm wavelength using a Tecan Infinite® M200 plate reader. Control wells containing untreated samples provided baseline measurements for comparative analysis (Kamel et al. 2018; Soliman et al. 2025).

#### Antioxidant capacity via DPPH radical scavenging procedure

For the antioxidant assessment, MgO NPs were tested at varying concentrations (15.7–1000 µg/mL) alongside a standard ascorbic acid solution. The procedure involved mixing MgO NPs with 1 mL of DH_2_O, followed by the addition of 1 mL of 1 mM DPPH (purity ≥ 95.0%, Sigma-Aldrich, St. Louis, MO, USA) solution. The reaction mixture was kept at room temperature for 0.5 h. before measuring absorbance at 517 nm using a UV–visible spectrophotometer (Periakaruppan et al. 2022).The DPPH radical scavenging activity (%) was then determined using (Eq. 1).

1 $$ {\text{DPPH Scavenged (\% )}} = \left( {\frac{{{\text{A}}_{{{\text{c}}0}} - {\text{A}}_{{{\text{s}}0}} }}{{{\text{A}}_{{{\text{c}}0}} }}} \right) \times 100 $$

whereas Ac0 is the absorbance of the control and As0 is the absorbance of the sample.

### Antidiabetic activity

#### α-Glucosidase inhibition

The inhibitory effect of MgO NPs on α-glucosidase was assessed using yeast-derived α-glucosidase and p-nitrophenyl-α-D-glucopyranoside (pNPG) (purity ≥ 99%, Sigma-Aldrich, St. Louis, MO, USA) as the substrate. Test samples and reference standards were prepared at suitable doses covering a range from 1000 to 15.625 μg/mL in PBS at suitable doses. The enzyme solution (0.1 M PBS, 1 U/mL) and substrate solution (10 mM pNPG) were prepared separately. For the assay, 100 μL of the enzyme solution was incubated at 37 °C for 20 min in test tubes, followed by the addition of 10 μL of pNPG solution and further incubation at 37 °C for 30 min. The enzymatic reaction was stopped by adding 650 μL of 1 M sodium carbonate solution. Absorbance was measured at 405 nm, and the inhibition percentages were calculated based on the absorbance readings (Kim et al. 2004).

#### Amylase inhibitory

Evaluation of α-amylase inhibition was carried out using the 3,5-dinitrosalicylic acid (DNSA) (≥ 98% purity, Sigma-Aldrich (St. Louis, MO, USA) assay. MgO NPs and reference standards were diluted in PBS at suitable doses covering a range from 1000 to 15.625 μg/mL. The assay mixture consisted of 10 μL of α-amylase solution (2 U/mL) and 20 μL of each test sample, incubated at 37 °C for 20 min. Then, 200 μL of a 1% potato starch solution in 100 mM PBS was introduced into each reaction tube, followed by an additional 30-min incubation at 37 °C. The reaction was terminated by adding 100 μL of DNSA reagent, after which the samples were heated at 90 °C for 10 min. After cooling to room temperature, absorbance readings were taken at 540 nm, and the inhibition percentage of α-amylase activity was determined (Wickramaratne et al. 2016).

### Statistical analysis

Data were analyzed using GraphPad Prism software (version 8; San Diego, CA, USA). Statistical comparisons between group means were performed using both one-way and two-way analysis of variance (ANOVA). Minitab® version 18 (2017) was used for optimization via RSM. All experimental results are presented as mean values ± SD. A value P with less than 0.05 was considered revealing of statistical significance.

## Results

### Phytochemical study

Screening of phytochemicals revealed that the OPE contains alkaloids, carbohydrate, flavonoid, glycosides, saponin, and tannin (Table S1).

### Optimization study for MgO NPs biosynthesize

#### Determination of significant factors

The maximal productivity of MgO NPs varies depending on environmental conditions, such as a presence of precursors concentration. The incubation time between the OPE filtrate and the precursors, along with pH and temperature, were the variables considered. The UV–Vis spectra of the biosynthesized precursors were used to identify them. From all four factors only, one factor exhibited non-significant outcomes among the bio fabrication of MgO NPs illustrated in the time of incubation between the OPE filtrate and precursors of MgO NPs (Fig. S1). So RSM was applied to identify the optimal parameters for maximizing the final MgO NPs productivity. The most important factors (precursor concentration, pH and temperature) were conducted to obtain the optimum condition for bio fabrication process.

#### RSM analysis

**Fig. 1:**
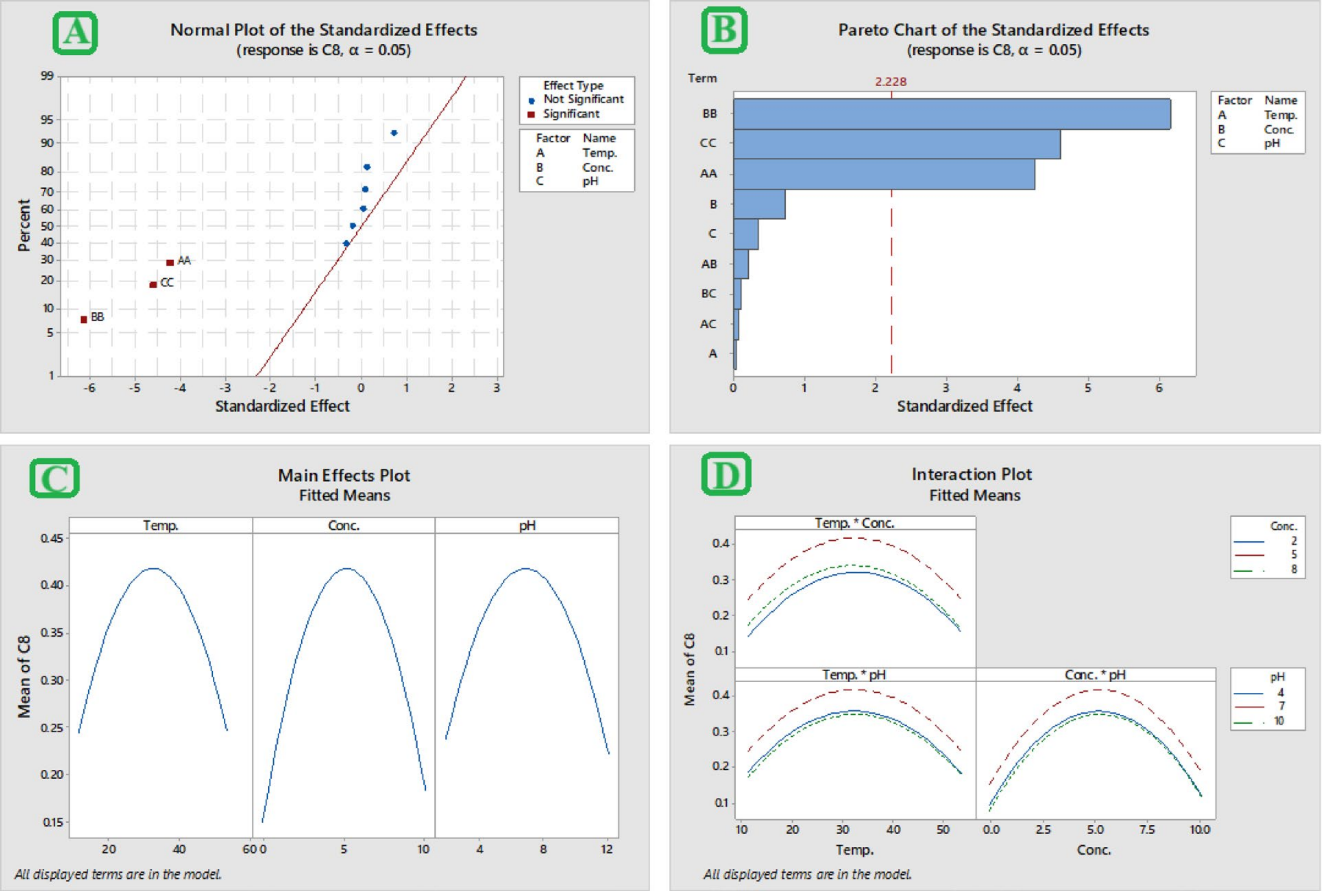
RSM analysis showing **A** Normal plot **B** Pareto chart **C** Main effects plot, and **D** Interaction Plot

**Fig. 2:**
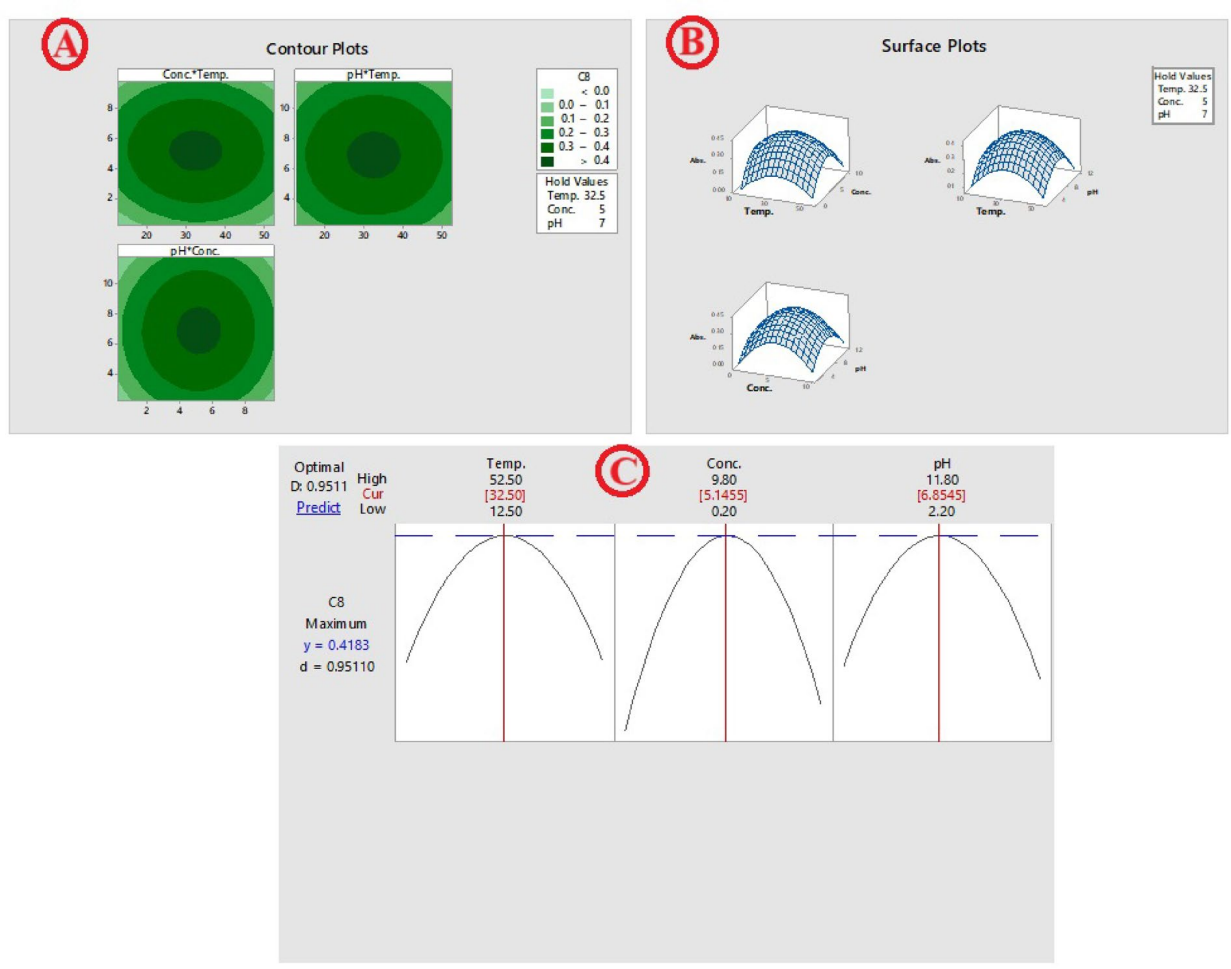
RSM analysis showing **A** Contour Plots **B** Surface Plots, and **C** Optimal predictor

To optimize the synthesis parameters for maximal yield and concentration of MgO NPs, RSM was utilized. The plot of normal probability MgO NPs show the true (real) effect of each term and whether chance (randomness) contributed to the outcomes. As illustrated in the normal probability plot (Fig. 1A), the direction of each factor's influence on the response—whether beneficial or adverse—is indicated by its position relative to the fitted line. A positive effect suggests that increasing a given variable enhances the response, whereas a negative effect implies a reduction in the response. Each interaction is illustrated as an individual point on the plot: points located further from the fitted line reflect variables with significant real effects, while those closer to the line indicate factors with negligible influence. Factors positioned on the right side of the plot contributed positively to MgO NP formation. Conversely, interactions such as concentration–pH (AA) and concentration–concentration (CC), found on the left side of the fitted line, demonstrated a significant negative impact on nanoparticle synthesis. A Pareto chart was utilized to assess the relative significance of both main effects and interaction terms in the experimental design (Fig. 1B). Pareto chart analysis conducted after 3 h revealed the most significant factors affecting MgO NPs biosynthesis. The primary impacts of temperature (A), concentration (B), pH (C), and their correlation to one another (AA, BB, CC, AB, AC, and BC), were indicated in a Pareto chart for MgO NPs synthesis. Main effect and interaction plots (Fig. 1C and D) were generated to illustrate the average differences between the maximum and minimum response values for each variable based on the predictive analysis outcomes. The findings revealed notable variations at the extreme levels of pH, concentration, and temperature. Surface and contour plots (Fig. 2A and B) were constructed to visually illustrate the influence of significant main and two-way interaction effects on MgO nanoparticle synthesis. Each plot displays the response behavior for two variables while the third factor, not shown in the graph, is held constant at its center level. These two-dimensional plots connect points of equal response values to form contour lines, ranging from the lowest to the highest nanoparticle yield. In addition, surface wireframe plots—three-dimensional graphical representations—were used to depict the relation between two interacting variables and the corresponding response. These 2D visualizations highlight the optimal regions (peaks) where the maximum nanoparticle production is observed. The results generated by the optimizer program were used to construct the optimization curves (Fig. 2D), which show the optimal settings for maximizing MgO NPs biosynthesis at pH 6.8, 32.5 °C, and 5.1 mmol were the ideal conditions for performing confirmatory experiments in triplicate, encompassing both preparation and incubation stages.

#### Characterization of MgO NPs using OPE at optimized conditions

**Fig. 3:**
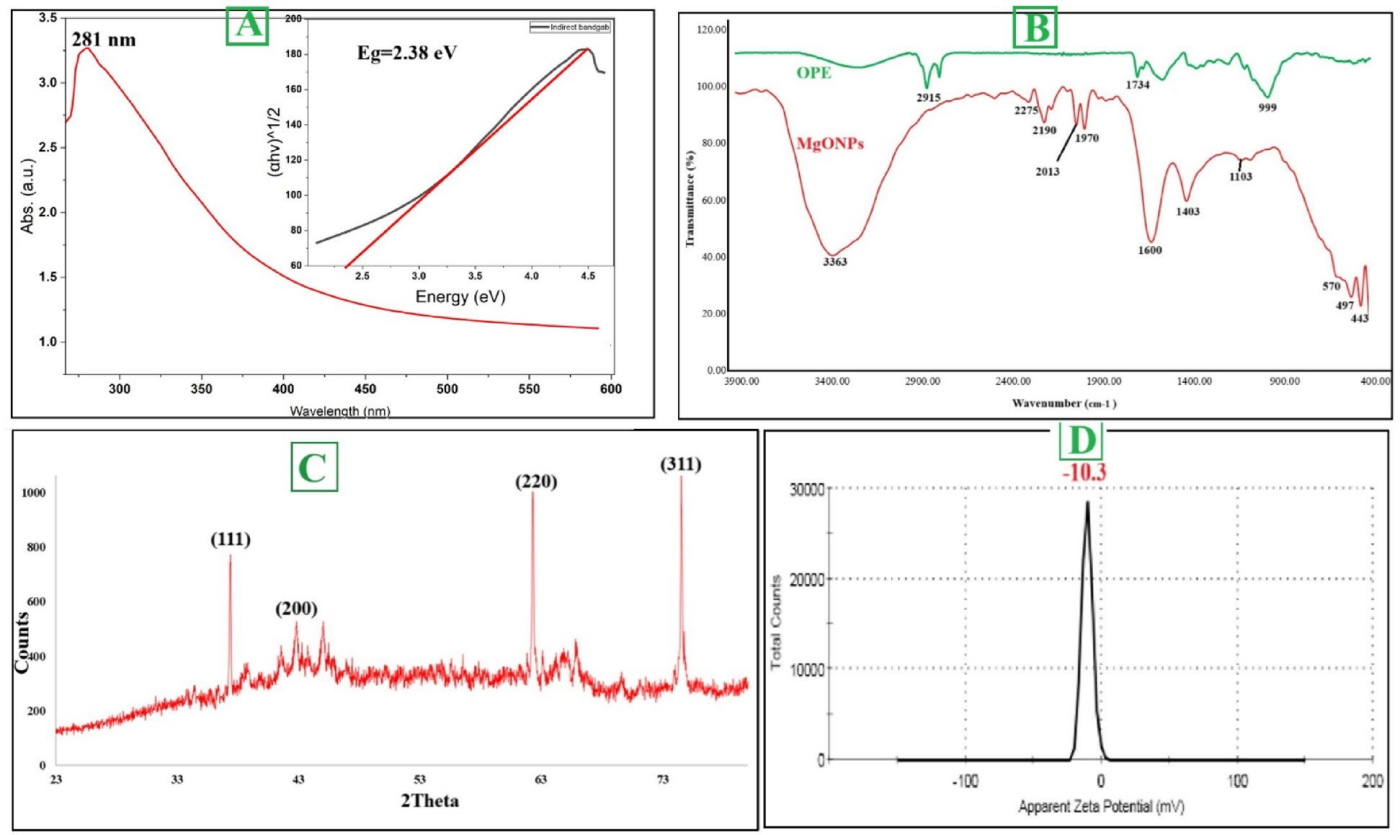
UV–Vis spectroscopy and Bandgap energy (**A**), FTIR (**B**), XRD (**C**), and Zeta potential (**D**) of MgO NPs

**Fig. 4:**
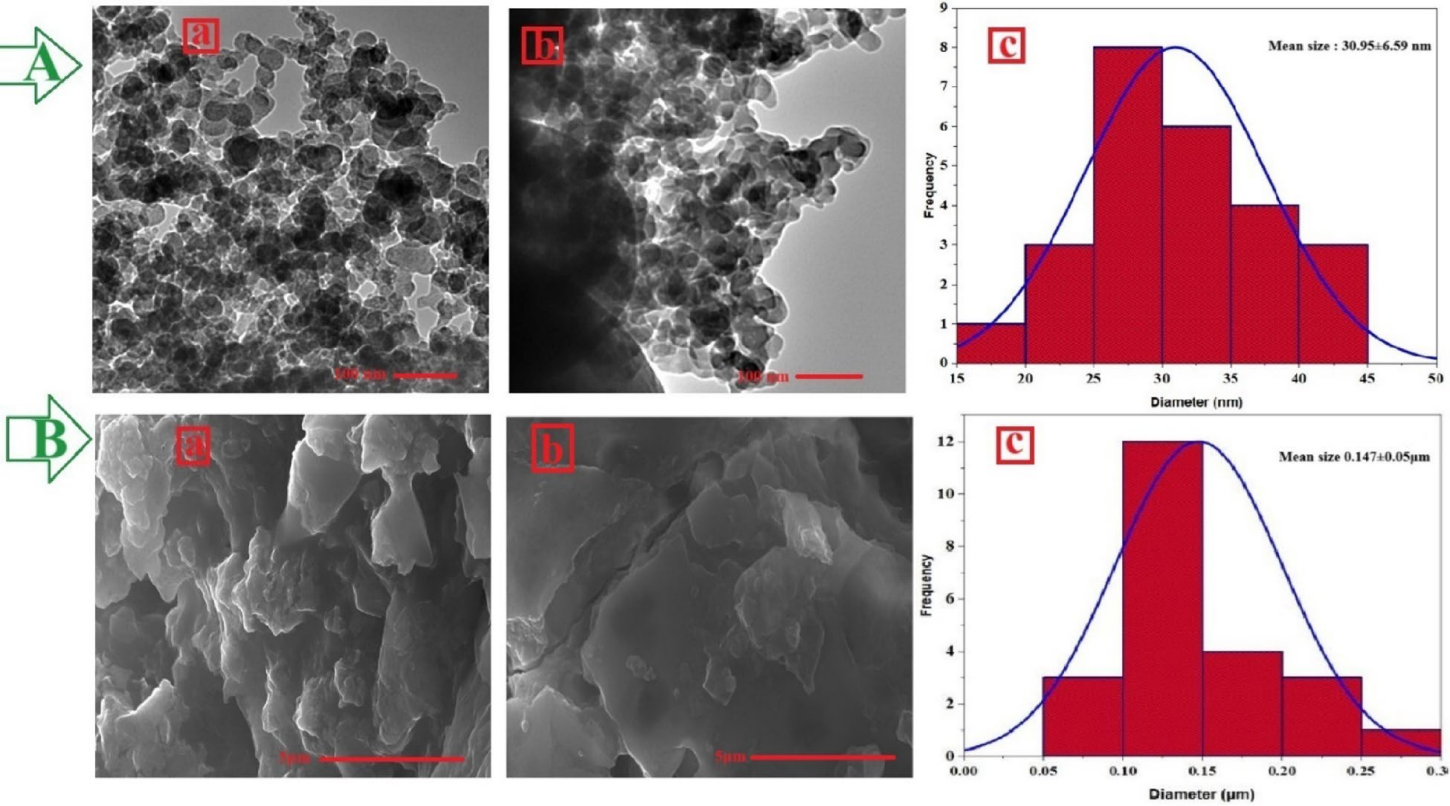
Morphological study TEM images A (**a**, **b**) distribution histogram (**c**) and SEM imagery B (**a**, **b**) distribution histogram(**c**) of MgO NPs

**Fig. 5:**
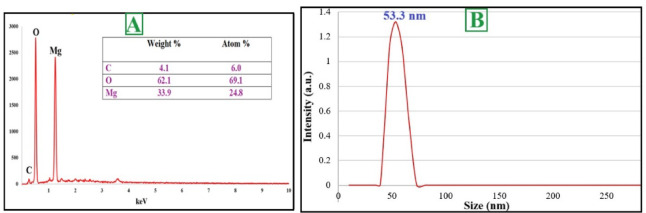
EDX study **A** and The DLS analysis **B** of bioinspired MgO NPs

Usage of OPE for MgO NPs biosynthesis remains largely unexplored, underscoring the innovation of the current study. The biosynthesize of MgO NPs had appeared by the color change that at the optimum condition for high productivity at pH 6.8, 32.5 °C, and 5.1 mmol. Table 1 provides a comparative analysis of the properties and bioactivities of the MgO NPs synthesized in this study against those reported in prior literature, encompassing both green and chemical synthesis methods. During the synthetic under optimization process, a well-defined surface plasmon resonance band emerged at 281 nm in UV–vis spectroscopy (Fig. 3A), demonstrating the typical optical absorption profile of MgO NPs. This SPR phenomenon results from the collective vibration of conduction electrons when exposed to electromagnetic radiation, and its detection confirms both successful nanoparticle formation and colloidal stability. The specific absorption maximum at 281 nm provides insight into the nanomaterial's photonic characteristics, which are determined by particle dimensions, shape, and chemical makeup. Further, the energy band gap was determined by Tauc plot analysis and the energy band gap for MgONPs prepared by OPE was 2.38 eV. FTIR analysis identified multiple bioactive compounds from the plant extract, including proteinaceous materials, saccharides, amino acid residues, and polymeric sugars, that participated in the ionic reduction process while simultaneously functioning as protective ligands for nanoparticle stabilization. The FTIR spectrum of the synthesized MgO NPs displayed distinct peaks at various wavenumbers, indicating the presence of diverse functional groups. As shown in (Fig. 3B), FTIR analysis of MgO NPs contains various peaks at wavenumbers of 3363, 2275, 2190, 2013, 1970, 1600, 1403, 1103, 570, 497, 443 cm^−1^. In addition to the FTIR of the OPE was had been the following peaks at wavenumber 2915, 1734 and 999 cm^−1^. Additional absorption bands observed in the spectrum further indicate the role of OPE-derived phytochemicals in reducing and stabilizing the nanoparticles during their formation. The crystalline properties and structural features of the MgO NPs synthesized using orange peel aqueous extract were examined by XRD analysis. The diffractogram revealed four prominent peaks corresponding to the 111, 200, 220, and 311 planes, appearing at 2-theta degrees of 37.39°, 42.82°, 62.27°, and 74.51°, respectively (Fig. 3C). XRD analysis revealed that the position and intensity of the observed peaks offer critical insights into the crystalline structure of MgO NPs. The appearance of sharp and well-defined peaks confirms that the plant-mediated MgO NPs possess a high degree of crystallinity with distinct crystallographic orientations. The average crystallite size was further estimated from the XRD data using the Debye–Scherrer equation applied to the most intense peaks. The calculated crystallite size was found to be 43.57 nm, which is consistent with the particle size distribution observed in DLS analysis, confirming the nanocrystalline nature of the synthesized MgO NPs. A zeta sizer of the synthesized MgO NPs was evaluated for stability and surface charge and the results revealed a value of − 10.3 mV (Fig. 3D). TEM was applied to investigate the morphological characteristics of the nanoparticles, specifically focusing on their size and shape. The synthesized MgO NPs exhibited semispherical morphology (Fig. 4A and B). In the TEM image, some aggregations of MgO NPs were identified. Similar aggregations were also evident in the SEM images (Fig. 5B), primarily attributed to surface interactions occurring at the nanoscale. As demonstrated, the aqueous extract of orange peel effectively facilitates the synthesis of well-organized, semispherical or quasi-spherical nanoparticles. The size and morphology of nanoparticles are crucial determinants of their functional activity. The scanning electron microscope (SEM) of MgO NPs derived from OPE reveals the morphology of these particles (Fig. 4B). The MgO NPs formed were predominantly semispherical or quasi-spherical in shape with some degree of agglomeration. The levels of phytochemicals in the extract primarily account for the bio-reducing and capping properties observed during synthesis which ultimately influence the shape of the particles. Particle size analysis (PSA) was performed by measuring individual nanoparticles from TEM and SEM micrographs. The MgO NPs were well-dispersed using ultrasonication prior to imaging. The size distribution histograms generated from this analysis (Fig. 4Ac for TEM and Fig. 4Bc for SEM) confirmed the nanoscale of the particles. The average particle size was calculated from the mean value of the histograms, yielding results of 30.9 nm from TEM analysis and 0.147 μm from SEM analysis. EDX analysis confirmed the elemental composition of the biosynthesized MgO NPs (Fig. 5A). Moreover, in (Fig. 3C) it shows that the primary constituents of MgO NPs were Mg and O with weight 33.9 and 62.1 while, atomic percentages 24.8 and 69.1% respectively. An essential approach for evaluating nanoparticle size distribution and colloidal stability involves the use of DLS. It allows for the distinction between monodisperse systems, where particles are uniform in size, and polydisperse systems, which exhibit a broad size range. In this study, the average particle size of the biosynthesized MgO NPs was found to be 53.3 nm (Fig. 5B). This result highlights the effectiveness of OPE in facilitating the formation of relatively small nanoparticles.

**Table 1:** Comparative analysis of MgO nanoparticles synthesized in this study versus other green and chemical methods

Synthesis Method	Size (nm)	Morphology	Biological Activities	References
Green: Orange Peel Extract	53.3	Spherical	Anticancer, Antibacterial, Antioxidant, and Antidiabetic	Current study
Green: *Sargassum wightii* (Seaweed)	33–92	Spherical & Oval	Anticancer and Antibacterial	Pugazhendhi et al. (2019)
Green: *Trigonella foenum-graecum* (Fenugreek)	40–50	Spherical	Antibacterial	Vergheese and Vishal (2018)
Green: *Rosa floribunda* (Flowers)	20–80	Spherical	Antibacterial and Antibiofilm	Younis et al. (2021)
Chemical: Co-precipitation	11	hexagonal	Antibacterial	Yadav et al. (2023)
Chemical: Co-precipitation	14–16	cubic	Antibacterial	Karthikeyan et al. (2016)
Chemical: Sol–gel	200–300	Spherical	–	Boddu et al. (2008)
Chemical: Solvothermal	18	Spherical	Antibacterial	Rukh et al. (2019)
Chemical: Combustion	20–35	flakes-like structures	Antibacterial	Tharani et al. (2021)
Physical/Chemical: Microwave-assisted	6—11	irregularly shaped	Antibacterial	Makhluf et al. (2005)
Chemical: Solvo- and Hydrothermal	100–200	Hexagonal	catalyst	Ding et al. (2001)

#### The anticancer activity of MgO NPs

**Fig. 6:**
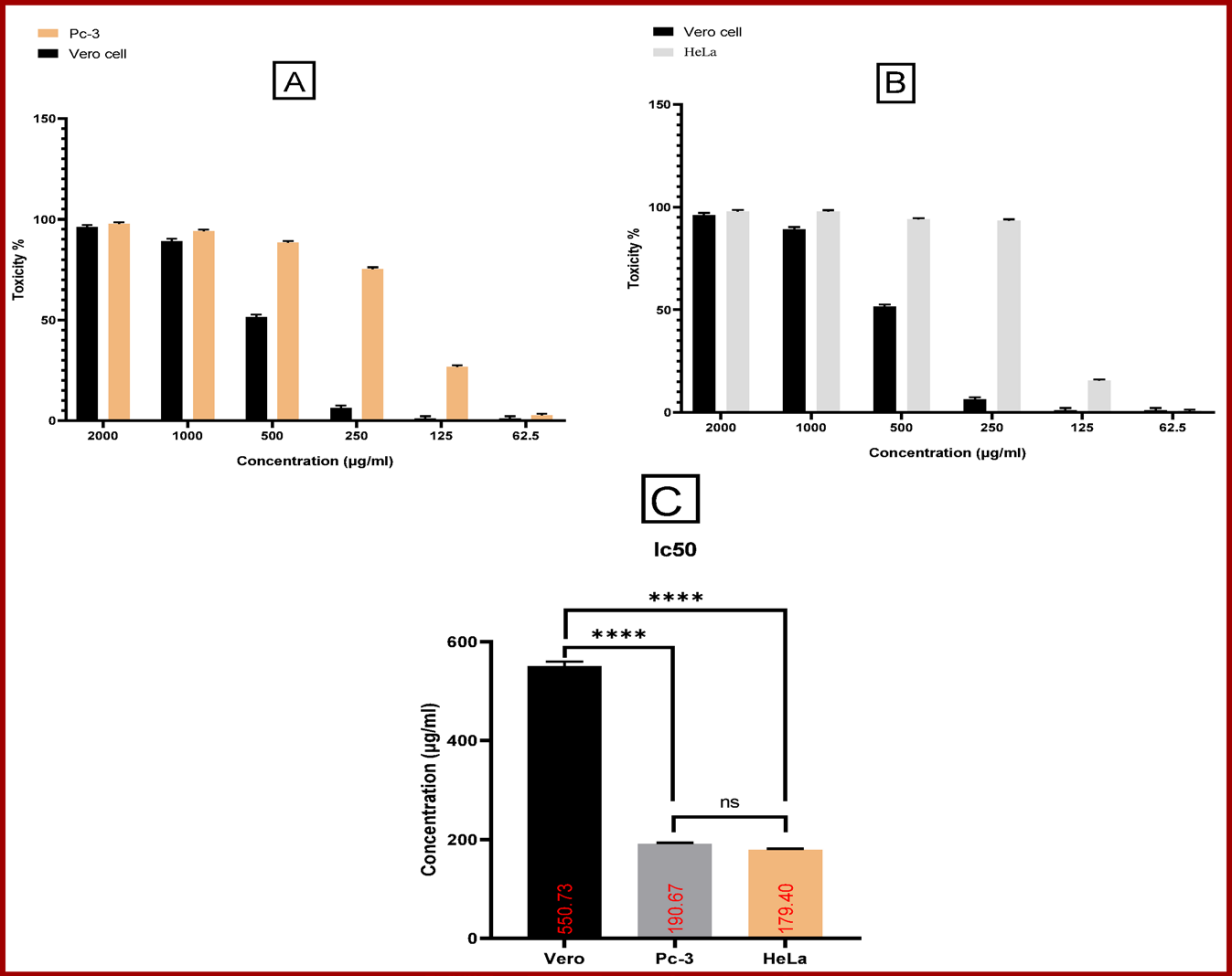
Viability, toxicity and IC50 of Vero, PC3 and HeLa cell lines after treated by MgO NPs

**Fig. 7:**
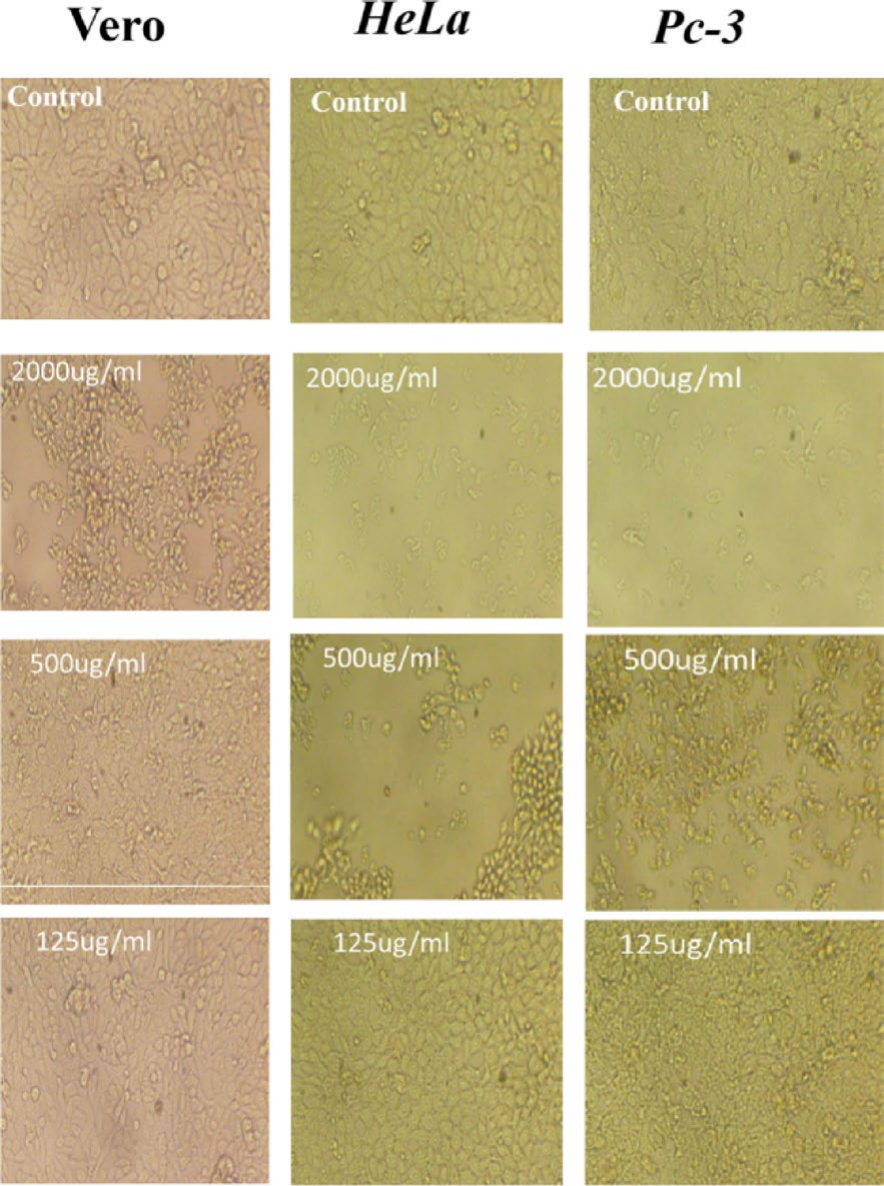
The morphology of Vero, PC3 and HeLa cell lines control and treated by bioderived MgO NPs

The cytotoxic effect of biosynthesized MgO NPs was first evaluated on the normal Vero cell line to assess general biocompatibility. As illustrated in (Fig. 6) the MgO NPs demonstrated low cytotoxicity against normal cells, with a high IC_50_ value of 550.7 μg/mL calculated across a concentration range of 2000 to 62.5 μg/mL (Fig. 6c). Subsequently, the anticancer potential of MgO NPs was assessed against two cancer cell lines prostate cancer (PC3) and cervical cancer (HeLa). The MgO NPs exhibited significant, dose-dependent cytotoxic effects against both cancerous cell lines (Fig. 6A, B). The IC_50_ values were determined to be 190.67 μg/mL for PC3 cells and 179.4 μg/mL for HeLa cells Fig. 6c, indicating a potent and selective anticancer effect compared to their minimal toxicity on normal Vero cells. To further elucidate the mechanism behind the observed cytotoxicity, we investigated the morphology of the cells via inverted microscope. As presented in (Fig. 7), shows cell death including both healthy and malignant cells significantly dose-dependent also when subjected to various quantities of MgO NPs.

#### Antibacterial and antibiofilm efficacy

**Fig. 8:**
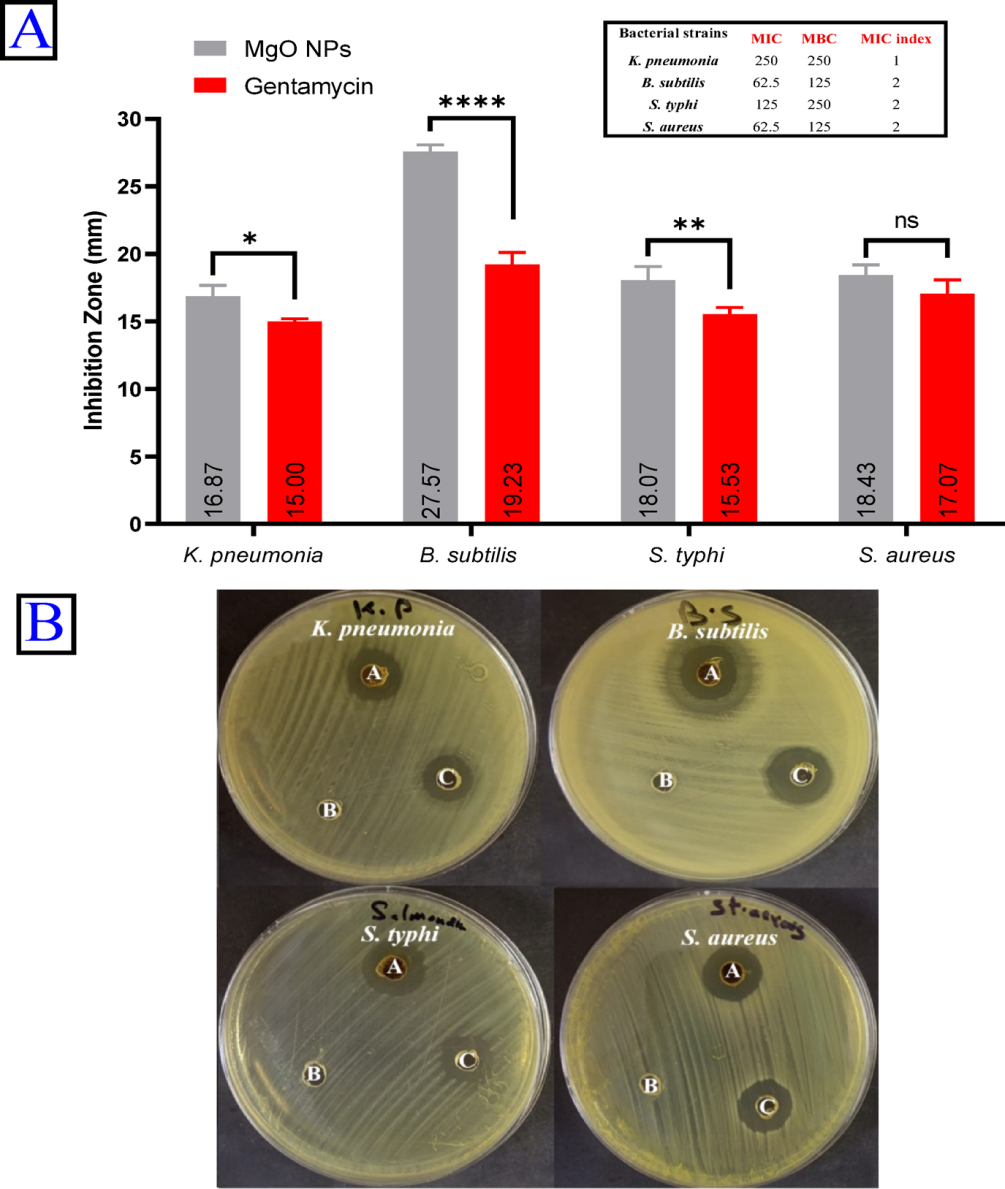
The antibacterial potentials of bioderived MgO NPs. A: Agar well diffusion and MIC index. B: petri plate of the bacterial strains (A: MgONPs, B: negative control (DMSO) and C: positive control (Gentamicin))

The rise of multi-drug resistant (MDR) microorganisms poses a significant threat to both the medical and agricultural sectors. These resistant strains have evolved sophisticated defense mechanisms that diminish the efficacy of conventional antibiotics, complicating treatment and management strategies. Consequently, researchers are intensively exploring innovative solutions, including the development of novel bioactive agents such as NPs. In this study, MgO NPs were biosynthesized using metabolites present in OPE which acted as both reducing and stabilizing agents to mediate the transformation of the metal precursor into MgO NPs. In this study, the antimicrobial efficacy of green-synthesized MgO NPs was evaluated against various pathogenic microorganisms, including *K. pneumonia* (ATCC 13883), *S. typhi* (ATCC 6539), *B. subtilis* (ATCC 6633) and *S. aureus* (ATCC 6538) (Fig. 8A, B). As illustrated, the greatest antibacterial effect was recorded at a high MgO NP concentration of 1000 μg/mL, producing zones of inhibition of 27.57 mm for *B. subtilis*, 18.43 mm for *S. aureus*, and 16.87 mm for *K. pneumonia*. In contrast, *S. typhi* exhibited sensitivity to the MgO NPs under the same conditions by 18.0 mm. Determining the MIC is a critical step in evaluating the effectiveness of bioactive compounds against pathogenic microorganisms. MIC refers to the lowest concentration needed to inhibit visible microbial growth. This measure provides essential insights into microbial susceptibility or resistance, supporting the appropriate selection and dosing of antimicrobial agents. In this study, the MIC values of green-synthesized MgO NPs were determined to be 62.5 μg/mL for *B. subtilis* and *S. aureus*, 125 μg/mL for *S. typhi*, and 250 μg/mL for *K. pneumoniae* (Fig. 8). Furthermore, the MIC index values were calculated as 1 for *K. pneumoniae* and 2 for *S. typhi, S. aureus* and *B. subtilis*. The antibiofilm potential of MgO NPs was evaluated against a range of bacterial isolates. Consequently, the strongest anti-biofilm effect of MgO NPs was observed against biofilms produced by *S. aureus* ATCC 35556 whenever employed at concentrations; 200, 100, 50, 25, 12.5, 6.25 and 3.12 µg/mL, the biofilm was reduced by 69.6, 66.8, 59.9, 51.6, 46.1, 39.0 and 26.7% respectively. Furthermore, MgO NPs showed strong decreasing for the biofilm of *E. coli* ATCC 25922 at the same doses while exerting no inhibitory effect on bacterial growth, with percentages of 55.5, 49.8, 44.7, 39.0, 28.0, 17.7 and 10.1%, accordingly (Fig. 5s).

#### Antioxidant activity

As illustrated in (Fig. S2), MgO NPs demonstrated notable antioxidant activity, compared to that of ascorbic acid, which served as the reference standard. The radical scavenging capacity of MgO NPs showed a dose dependent increase across the tested concentrations. The IC_50_ values were calculated as 180.2 μg/mL for MgO NPs and 28.49 μg/mL for ascorbic acid. Although the MgO NPs exhibited lower antioxidant potency than the standard, they retained significant free radical scavenging activity. This activity may be attributed to the presence of bioactive constituents within the OPE extract.

#### Antidiabetic activity

The antidiabetic potential of MgO NPs is assessed by measuring their ability to inhibit α-amylase and α-glucosidase enzymes. Inhibiting α-amylase and α-glucosidase is a crucial therapeutic strategy in managing type 2 diabetes, as these enzymes significantly contribute to postprandial hyperglycemia. In this study, α-glucosidase inhibition was quantitatively assessed using a spectrophotometric assay involving yeast α-glucosidase and p-nitrophenyl-α-D-glucopyranoside (pNPG) following treatment with varying concentrations of MgO NPs and the reference drug acarbose (Fig. S3). MgO NPs exhibited dose-dependent α-glucosidase enzyme inhibition, with an IC_50_ of 38.8 μg/mL, while10.52 μg/mL for acarbose. The observed enhancement in activity was attributable to the pronounced antioxidant properties of the MgO NPs. A comparable trend was observed in the α-amylase inhibition assay (Fig. S4), where MgO NPs showed an IC_50_ of 76.71 μg/mL, while acarbose demonstrated higher potency with an IC_50_ of 27.89 μg/mL.

## Discussion

The sustainable synthesis of biogenic MgO NPs using OPE represents a significant advancement in eco-friendly nanotechnology, as it merges green chemistry principles with biomedical innovation. Phytochemical analysis of the OPE revealed the presence of alkaloids, tannins, flavonoids, saponins, carbohydrates, and glycosides. The detected alkaloid content suggests that the extract possesses notable medicinal properties. Furthermore, our detection of phenolic compounds aligns with previous research by (Koolaji et al. 2020), which examined the phenolic profiles of agricultural byproducts, including orange peels, pea peels, and rice husks. That study reported that orange peels had the highest total phenolic content, a finding which strongly supports our results. The phytochemicals present in orange peel extract particularly flavonoids, alkaloids, tannins, and phenolic compounds play a dual role in the green synthesis of MgO NPs, serving as both reducing and capping agents. Reduction of Mg^2^⁺ ions as flavonoids and phenolic acids act as natural reducing agents. Their hydroxyl (–OH) and carbonyl (C=O) groups donate electrons to convert Mg^2^⁺ to Mg⁰ nuclei, a mechanism reported in other plant-mediated syntheses (Pugazhendhi et al. 2019). Similarly, alkaloids contribute to the reduction process via their amine groups, which stabilize intermediate complexes during nucleation (Younis et al. 2021). For stabilization of NPs, the carboxyl (–COOH) and hydroxyl (–OH) groups of OPE phytochemicals bind to MgO surfaces, forming a protective layer that prevents aggregation. This capping role is confirmed by FTIR peaks observed at 1655 cm^−1^ (C=O) and 3444 cm^−1^ (–OH) (El-Naggar et al. 2020) (Alaizeri et al. 2021). The successful production of green nanoparticles is contingent upon various parameters, including reaction pH, incubation temperature, synthesis duration, precursor concentration, and the chosen synthesis approach (Abu-Elghait et al. 2025). Optimizing these parameters is essential for maximizing growth and yield in biotechnological processes (Kuhl et al. 2020).

In our study, we employed Response Surface Methodology (RSM) to design experiments and determine the optimal conditions for MgO NP production. This technique allowed us to systematically evaluate the most effective factors: pH, temperature, and precursor concentration. The RSM analysis yielded several key outcomes. For instance, a normal probability plot of residuals was used to detect significant deviations from normal distribution assumptions (Bagewadi et al. 2018). This finding aligns with other studies confirming that concentration, pH, and the protective agent significantly influence the size of the resulting MgO NPs (Draper and Smith 1998; Nyakundi and Padmanabhan 2015). Furthermore, main effects plots specifically illustrated how changes in each individual parameter influence nanoparticle synthesis, demonstrating their respective contributions to the overall response (Jiang et al. 2022). The efficacy of this statistical approach is supported by a related study on silver nanoparticles (Barabadi et al. 2019). Using a Box-Behnken Design, that study identified key influencing parameters and found a strong correlation between predicted and observed values (R^2^ = 0.8894), concluding that optimization offers a cost-efficient and time-saving approach. Ultimately, through RSM, the optimal condition for MgO NP biosynthesis was determined to be pH 6.8, a temperature of 32.5 °C, and a precursor concentration of 5.1 mmol.

Under the optimized synthesis conditions, the bio fabricated MgONPs exhibited a characteristic ultraviolet–visible absorption peak at 281 nm. This finding is consistent with previous studies reporting surface plasmon resonance maxima for MgO NPs within the 260–280 nm range (Moorthy et al. 2015; Vergheese and Vishal 2018). Specifically, the most intense SPR peak for MgONPs biosynthesized from *Tecoma stans* (L.) was also recorded at an identical wavelength of 281 nm (Nguyen et al. 2021). The position of the SPR peak is correlated with particle size, wherein smaller nanoparticles are associated with absorption at shorter wavelengths (< 300 nm) (Jeevanandam et al. 2017). The calculated band gap energy (E_g_) of 2.38 eV is significant, as it confirms the semiconducting nature of the synthesized MgO nanoparticles, indicating optical activity primarily within the UV range (Kadham et al. 2018). FTIR spectroscopy was used to identify the functional groups involved in the synthesis and capping of the nanoparticles. The FTIR spectrum of the OPE revealed key peaks at 2915, 1734, and 999 cm^−1^. The peak at 2915 cm⁻^1^ is attributed to C–H stretching vibrations in aliphatic hydrocarbons, commonly found in long-chain fatty acids and terpenes (Kumar Sahu et al. 2019). The strong peak at 1734 cm^−1^ corresponds to the C=O stretching vibration of carbonyl groups in esters and carboxylic acids, indicative of pectin, flavonoids, and citric acid present in the peel (Kute et al. 2020). The peak at 999 cm^−1^ is likely due to C–O stretching vibrations or glycosidic linkages in carbohydrates (Sutradhar and Saha 2016). The FTIR spectrum of the phytosynthesized MgO NPs showed different peaks at 3363, 2275, 2190, 2013, 1970, 1600, 1403, 1103, 570, 497, and 443 cm^−1^. The broad peak at 3363 cm^−1^ is due to O–H stretching from absorbed water and phenolic compounds capping the nanoparticles, with its broadness indicating strong hydrogen bonding (Jeevanandam et al. 2022). The peaks at 1600 cm^−1^ and 1403 cm^−1^ are crucially important and are assigned to the asymmetric and symmetric stretching vibrations of carboxylate ions (COO⁻), respectively. This suggests carboxylic acids from the OPE donated protons during reduction, forming carboxylate anions that now stabilize the MgO nanoparticle surface (Mg⁺–OOC–R) (Chandrasekaran et al. 2023). The peak at 1103 cm^−1^ can be attributed to C–O stretching in alcohols, phenols, or ethers of capping agents (Shende et al. 2015). Peaks in the region 2275–1970 cm^−1^ may be attributed to O=C=O or C≡N stretching. Finally, the low-frequency peaks at 570, 497, and 443 cm^−1^ are definitive fingerprints of Mg–O stretching vibrations, confirming the formation of the periclase crystal structure of MgO (Jeevanandam et al. 2022, 2017; Muhaymin et al. 2024). X-ray diffraction analysis further confirmed the crystallinity of the synthesized MgO NPs. The diffractogram revealed four prominent peaks corresponding to the (111), (200), (220), and (311) planes at 2θ values of 37.39°, 42.82°, 62.27°, and 74.51°, respectively. This pattern is in agreement with the standard Joint Committee on Powder Diffraction Standards (JCPDS file no. 75-0447) (Essien et al. 2020). This result is consistent with other bio-synthesis studies, such as that using *Rosa floribunda* extract (Younis et al. 2021). The colloidal stability of the bio fabricated MgONPs was assessed by zeta potential measurement, which yielded a value of − 10.3 mV, indicating a moderately stable formulation (Amina et al. 2020). Morphological analysis via TEM and SEM revealed that the MgONPs were predominantly semispherical or quasi-spherical in shape, with some degree of agglomeration. This agglomeration is common in green synthesis and can be attributed to surface-bound biomolecules that facilitate interactions between particles (Arabi et al. 2020; Shanavas et al. 2020). These findings are consistent with the work of (Dabhane et al. 2022), who observed comparable morphology for MgO NPs synthesized using *Ajwain* leaf extract. Energy-dispersive X-ray spectroscopy confirmed the elemental composition of the nanoparticles. The main components were magnesium and oxygen, with weight percentages of 33.9% and 62.1%, and atomic percentages of 24.8% and 69.1%, respectively. This aligns with the expected composition of MgO and is supported by similar findings in other studies (Younis et al. 2021).

In this work, the average particle size of the biosynthesized MgO NPs was found to be 53.3 nm. Assessing the experimental cytotoxicity of biological materials against normal cell lines represents a fundamental initial step in evaluating their safety profile (Salem and Fouda 2021). Our result revealed that the IC_50_ values were 190.67 μg/mL for PC3 cells and 179.4 μg/mL for HeLa cells, indicating a dose-dependent cytotoxic response. This result aligns with the findings of (Patel et al. 2013), who reported that the cytotoxic effects of MgO NPs are dose-dependent. Their study further suggested the potential application of MgO NPs in anticancer therapies. The mechanism of cellular uptake, often via endocytosis, can be influenced by the nanoparticles' physicochemical properties. Our synthesized MgO NPs exhibited a zeta potential of -10.3 mV. Although this initial charge is slightly negative, it can be modulated in biological media by the formation of a protein corona, and uptake is a complex process influenced by multiple factors including size, shape, and cell type (Kandiah et al. 2019). The strong anticancer efficacy is further supported by other studies; for instance, an MTT assay on HeLa cells showed significant growth inhibition at concentrations of 25–75 μg/mL, with an IC_50_ of 56.54 μg/mL (Sambandam et al. 2025). Furthermore, while other nanocomposites like FeO_4_–RGO show biocompatibility (Ahamed et al. 2020), and bimetallic particles like Ag–MgO show enhanced cytotoxicity (Saravanakumar and Wang 2019), our green-synthesized MgO NPs demonstrate significant efficacy on their own. Also, (Wang et al. 2013) demonstrated the cytotoxic ability of chitosan-magnetic graphene nanoparticles and reduced graphene oxide against human PC3 cell lines. The green synthesis approach itself offers advantages over conventional methods, including enhanced sustainability, reduced use of toxic reagents, lower energy consumption, and improved biocompatibility (Alsaiari et al. 2023). In this study MgO NP concentration of 1000 μg/mL, producing inhibition zones of 27.57, 18.43, 16.87 and 18.0 for *B. subtilis, S. aureus*, *K. pneumonia* and *S. typhi* respectively*.* Furthermore, MIC data play a vital role in minimizing the development of antibiotic resistance, comparing the antimicrobial potency of different substances, guiding novel therapeutic approaches, informing drug formulation, and contributing to evidence-based clinical decision-making and the design of effective treatment strategies (Kowalska-Krochmal and Dudek-Wicher 2021). The MIC index values were determined to be 1 for *B. subtilis* and 2 for *S. aureus*, *K. pneumonia* and *S. typhi.* While MIC was 62.5, 250 and 125 μg/mL for *B. subtilis, S. aureus*, *K. pneumonia* and *S. typhi* respectively*.* The results indicated that antibacterial efficacy was positively correlated with concentration, aligning with findings reported in several previous studies (Vergheese and Vishal 2018). (Nguyen et al. 2018) observed that the adhesion behavior of pathogenic microorganisms such as *Pseud. aeruginosa*, *Staph. aureus*, methicillin-resistant *Staph. aureus* (MRSA), and yeasts such as *Can. albicans* and *C. glabrata* was enhanced at higher MgO NP concentrations (1400 and 1600 μg/mL), while lower concentrations resulted in reduced adhesion. Their antibacterial action is attributed to multiple mechanisms, including the alteration of membrane structural integrity. The nanoscale size and high surface area of the green-synthesized MgO NPs enhance their binding to bacterial membranes, leading to damage of the lipid bilayer and alteration or loss of selective membrane permeability (Alsaiari et al. 2023). A primary mechanism is the generation of reactive oxygen species (ROS)—such as superoxide radicals, hydrogen peroxide, and hydroxyl radicals—which induce oxidative stress that overwhelms bacterial defense systems (Chandraker and Kumar 2024). The porous, anionic nature of the peptidoglycan wall in Gram-positive bacteria like *B. subtilis* facilitates a strong electrostatic attraction with the nanoparticles, enhancing their activity (Krishnamoorthy et al. 2012; Wang et al. 2017). In our study, the biosynthesized MgO NPs exhibited significant antibiofilm activity against *S. aureus* ATCC 35556 and *E. coli* ATCC 25922. Crucially, this effect occurred without inhibiting the growth of the planktonic bacteria, indicating a specific action against the biofilm matrix itself rather than a general biocidal effect. This finding is consistent with a growing body of research on MgO NPs. For instance, (Nguyen et al. 2018) reported similar antibiofilm effects using *Staphylococcus epidermidis* as a model organism. Furthermore, (Younis et al. 2021) also documented the antibiofilm potential of green-synthesized MgO NPs. A study by (Shankar et al. 2023) demonstrated that MgO NPs (at 10, 20, and 40 µg/mL) significantly inhibited biofilm formation in *Bacillus cereus* and *Pseudomonas aeruginosa* in a dose-dependent manner, with a more pronounced effect observed against *B. cereus*. The existence of ROS has been connected to several chronic illnesses, including cancer, diabetes mellitus, and brain dysfunction. Elevated reactive oxygen species (ROS) levels are associated with age-related conditions such as cataracts, cardiovascular diseases, and cellular aging (Zimmerman et al. 2015). To counteract these harmful effects, bioactive compounds known as antioxidants neutralize free radicals and ROS, supporting cellular health and reducing disease risk (Kumar et al. 2016). In experimental studies, MgO NPs showed considerable antioxidant capacity, with IC_50_ values of 180.2 μg/mL, compared to ascorbic acid's 28.49 μg/mL, aligning with prior research on their antioxidative effects (Sharmila et al. 2019). Plants combat oxidative stress by producing secondary metabolites, particularly phenolics, which help mitigate ROS damage (Kumar et al. 2020). These phenolic compounds also play a key role in nanoparticle biosynthesis, facilitating the reduction of metal ions and subsequent functionalization with antioxidant properties (Sharma et al. 2021). The management of postprandial hyperglycemia is a critical strategy in diabetes care, primarily targeting the enzymes α-amylase and α-glucosidase. These enzymes catalyze the breakdown of complex carbohydrates into absorbable glucose units (Forman and Zhang 2021). Our biosynthesized MgO NPs exhibited potent, dose-dependent inhibitory activity against these key enzymes. The calculated IC_50_ values were 76.71 μg/mL for α-amylase and 38.8 μg/mL for α-glucosidase. While these values are higher than those of the standard drug acarbose (27.89 μg/mL and 10.52 μg/mL, respectively), they demonstrate significant potential, especially given the NPs' multi-functional nature. This antidiabetic effect is particularly relevant given the link between hyperglycemia and oxidative stress. Elevated blood glucose levels promote oxidative damage through glucose auto-oxidation and free radical formation, which significantly contributes to the pathogenesis of diabetes and its complications (Caturano et al. 2023). The observed enzyme inhibition, coupled with the inherent antioxidant activity of the NPs, presents a dual therapeutic approach. The multifaceted biological activities of the MgO NPs—including antidiabetic, antioxidant, antimicrobial, and anticancer effects—are intrinsically linked to their unique physicochemical properties (Wang et al. 2017). Their nanoscale size (53.3 nm) provides a high surface-area-to-volume ratio, maximizing contact with biological membranes and increasing reactive sites for interaction (Jeevanandam et al. 2018). Their semispherical morphology influences cellular uptake dynamics, while their surface charge (zeta potential of − 10.3 mV) can be modulated in biological media by protein corona formation. This modulation ultimately determines their electrostatic interactions with negatively charged microbial and cellular membranes (Wahab et al. 2014). Collectively, these properties govern the key mechanisms of action, such as reactive oxygen species (ROS) generation, metal ion release, and direct membrane disruption (Sirelkhatim et al. 2015), explaining their broad-spectrum efficacy.

In discussing the broader implications of our findings, it is crucial to emphasize their specific relevance to disability health. The multifunctional bioactivities of the biosynthesized MgO NPs, spanning anticancer, antimicrobial, antioxidant, and antidiabetic effects, converge to address a constellation of health challenges that are notably prevalent within disability communities. The high efficacy against wound associated pathogens like *S. aureus* and *E. coli*, coupled with significant antibiofilm action, suggests immense potential for managing pressure ulcers and surgical site infections, which are common and serious complications for individuals with limited mobility (Mirhaj et al. 2022). The antidiabetic and antioxidant properties further offer a therapeutic avenue for managing metabolic syndromes often associated with certain disabilities or their treatments (Robertson et al. 2015). Therefore, this study transcends fundamental nanomedicine; it signifies a strategic advancement towards inclusive, multi-purpose therapeutics designed to alleviate the complex and interconnected health disparities faced by individuals with disabilities, a key priority in modern healthcare research (Hetta et al. 2023).

### Limitations

Problems with targeted delivery: It might be challenging to deliver medication precisely to certain tissues, which can result in systemic toxicity and adverse effects. Toxicity observed despite low-risk profile: while MgO NPs are often less harmful than other nanoparticles, certain research indicates that they can nevertheless be highly harmful both in vivo and in vitro. Unreliable synthesis approaches: poor batch-to-batch repeatability and inconsistent methodologies plague industrial-scale production. Problems with scalability and polydispersity: because nanoparticles frequently differ in size and shape, it might be difficult to produce them uniformly on a large scale. Limited stability in the long run data: little is known about the long-term safety, behavior and stability of MgO NPs in human body. Absence of validated in vivo models: the models that are now available are insufficient for assessing the effects of treatments and interactions of the immune system, particularly immunotoxicity. Underdeveloped industrial infrastructure: A lack of tools and quality control systems hinders the shift from laboratory-scale research to industrial output. Clinical translation challenges: There are still some unresolved scientific and regulatory obstacles that prevent promising lab results from being successfully translated into clinical settings.

### Futuristic studies

Mechanistic studies: Examine the molecular mechanisms by which MgO NPs produce their antibacterial and therapeutic properties. In-vivo testing and biocompatibility: To assess the safety, adverse effects, and suitability of MgO NPs for biological systems, additional animal research is needed. Enhancing theranostics: Create theranostic systems based on MgO NPs for integrated diagnosis and treatment, an area that is still in its infancy. Potential for personalized medicine: Make use of MgO NPs to provide individualized care by utilizing their anti-inflammatory and antioxidant qualities to fit the needs of certain patient profiles. Integration of AI and Machine Learning: Use AI/ML to forecast optimal experimental conditions, optimize the synthesis of MgO NPs, and create nanoparticles with desired characteristics. Frameworks for regulations and standardization: To facilitate clinical translation, clearly define production, safety, and pharmacological profiling guidelines. Examine the possibilities for commercialization and patenting of MgO NPs in clinical, environmental, and industrial contexts.

## Conclusion

Orange peel extract was successfully used to synthesis MgO NPs, providing a safe, economical, environmentally beneficial, and green method. Characterization through FTIR, UV–Vis, XRD, Zeta potential, TEM, SEM–EDX, and DLS confirmed that the nanoparticles were approximately 53.3 nm in size, exhibited a semispherical or quasi-spherical and well-organized morphology, and possessed a crystalline structure. Furthermore, the presence of various phytochemical components within the OPE was confirmed, which played a crucial role in the reduction and stabilization of the nanoparticles. Then, RSM was used to find the highest productive MgO NPs. These biosynthesized MgO NPs displayed potent anticancer activity against HeLa cervical cancerous cell line and prostate cancerous cell line (PC3), where the IC_50_ was 190.67 and 179.4 μg/mL correspondingly. Also, MgO NPs show antibacterial activity toward various bacteria with highly inhibition of biofilm of *S. aureus* ATCC 35556 and *E. coli* ATCC 25922. Additionally, they exhibited remarkable antioxidant effects by DPPH assay and IC50 was 180.2 μg/mL. The MgO NPs antidiabetic efficacy was assessed based on their repressing impacts on α-amylase and α-glucosidase enzymes and compared to the reference drug acarbose. This comparison underscores the promising potential of MgO NPs in the management of diabetes through enzymatic regulation. This research introduces multifunctional MgO nanoparticles as a pivotal, biocompatible advancement to directly address the complex comorbidities faced by the disability community, consolidating multiple therapeutic properties into a single agent to mitigate polypharmacy and improve quality of life.

## Supplementary Information

Additional file1 (DOCX 160 kb)

## Data Availability

All data that support the findings of this study are available within the article.
