# Supplementary material for: Ecofriendly magnesium oxide nanoparticles: anticancer, antimicrobial, and antidiabetic potentials in vitro
Source: AMB Express. 2025 Oct 3;15:143. doi: 10.1186/s13568-025-01950-1 (PMC12494522; doi:10.1186/s13568-025-01950-1)
Supplement: Supplementary file 1 — Additional file1 (DOCX 160 kb) [file 13568_2025_1950_MOESM1_ESM.docx]

Table S1: Phytochemical study of OPE filtrate

| **Items** | **Phytochemicals** | **Availability** |
| --- | --- | --- |
| 1 | Carbohydrate | ++ |
| 2 | Alkaloids | + |
| 3 | Flavonoid | + |
| 4 | Glycosides | + |
| 5 | Saponin | + |
| 6 | Tannin | + |
| 7 | Phenols | - |
| 8 | Quinones | - |
| 9 | Steroids | - |

++ moderately, + weak, - completely absent

Figure 1: General factors for selecting the most significant ones by OVAT

Figure 2: The antioxidant efficacy of bioderived MgO NPs via DPPH assay.

Figure 3: The antidiabetic efficacy of bioderived MgO NPs upon α-glucosidase enzyme.

Figure 4: The antidiabetic efficacy of bioderived MgO NPs upon α amylase enzyme

Figure 5: Biofilm inhibition by bio generated MgO NPs against *S. aureus* ATCC 35556

and *E. coli* ATCC 25922
